# The Emerging Role of Transcription-Associated Cyclin-Dependent Kinases in Gastrointestinal Tumors

**DOI:** 10.3390/cancers18060979

**Published:** 2026-03-18

**Authors:** Dipti Athavale, David Pulipati, Curt Balch, Junsong Zhao, Yanting Zhang, Xiaodan Yao, Shumei Song

**Affiliations:** 1 Coriell Institute for Medical Research, 403 Haddon Ave., Camden, NJ 08103, USA; 2 MD Anderson Cancer Center at Cooper, Cooper University Hospital, 2 Cooper Plaza, Camden, NJ 08103, USA; 3 Departments of Biomedical Sciences, Cooper Medical School of Rowan University, 401 Broadway, Camden, NJ 08103, USA

**Keywords:** transcription-associated cyclin-dependent kinases, gastrointestinal cancers, RNA polymerase II, inhibitors, super enhancer, toxicity, resistance

## Abstract

Transcription-associated cyclin-dependent kinases (tCDKs) precisely control the gene transcription process (initiation, elongation, and termination) by mediating RNA polymerase II phosphorylation. In several cancers, disrupted transcriptional control is emerging as a hallmark. In this review we summarize research studies of tCDKs’ role in gastrointestinal (GI) tumors, particularly, in the biology of esophageal, gastric, pancreatic, and hepatobiliary cancers. Across these tumor types, tCDKs are implicated as activators of super enhancer (SE) regions and contribute to the “transcriptional addiction” that not only drives cancer cell growth but is also attributed to therapeutic vulnerabilities. Overall, expression of tCDKs is increased in GI tumor tissues, indicating a rational target for therapeutics. We further describe emerging approaches, including genetic manipulation, small-molecule inhibitors or targeted protein degradation that disrupt tCDK functions in GI malignancies. We conclude by describing key challenges in targeting tCDKs and future treatment directions.

## Simple Summary

Transcription-associated cyclin-dependent kinases (tCDKs) play critical roles in regulating RNA polymerase II activity and gene transcription. Many cancers, including gastrointestinal (GI) tumors, become unusually dependent on enhanced-transcriptional activity (“transcriptional addiction”) that promotes cancer cell proliferation, survival, and aggressiveness. This review summarizes the cancer-specific functions and clinical relevance of tCDKs (including CDK7, CDK8, CDK9, CDK10, CDK11, CDK12, CDK13, and CDK19), with a particular focus on GI tumors (esophageal, gastric, pancreatic, and hepatobiliary cancers). We further summarize preclinical studies and clinical trials evaluating therapeutic strategies targeting tCDKs, including small-molecule inhibitors, degraders, and genetic approaches, in GI tumors. Finally, we highlight key knowledge gaps and challenges in targeting tCDKs and suggest novel strategies to disrupt tCDK function, including rational drug combinations and their effects on antitumor immunity.

## 1. Introduction

### 1.1. Transcription-Associated CDKs (tCDKs)

**Figure 1:**
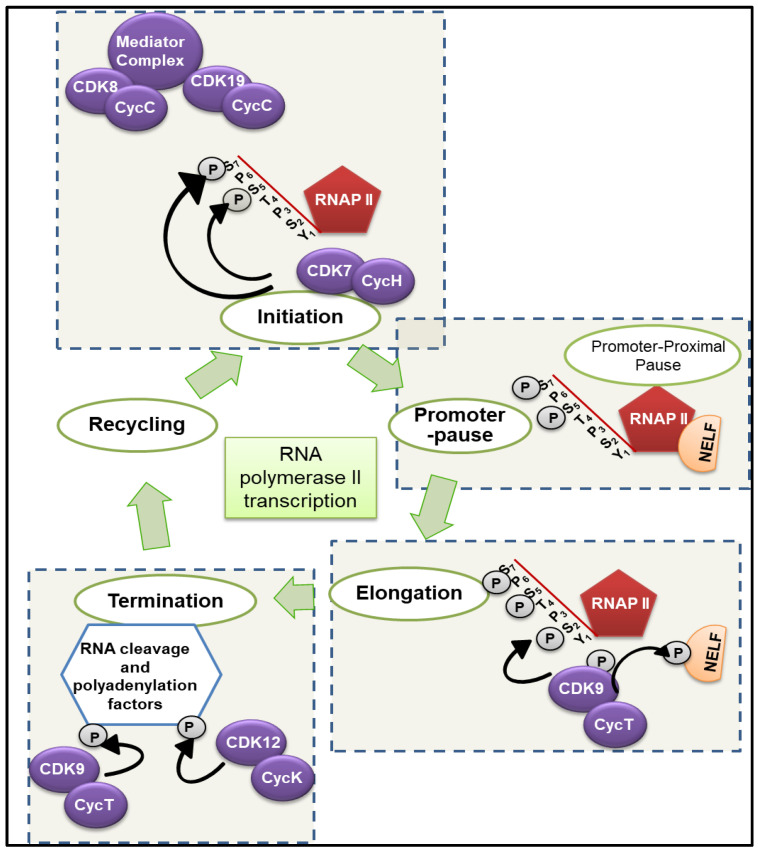
Role of tCDKs in RNAP II-mediated transcription. A CDK-activating kinase (CAK) complex comprising CDK7, Cyclin H (CycH), and MAT1 forms a part of the pre-initiation complex (PIC) that assembles at promoter regions and phosphorylates RNAPII-CTD-ser5 and RNAPII-CDT-ser7 residues to initiate RNA transcription. CDK8 and CDK19 are a part of the mediator complex and regulate RNAP II activity both positively and negatively at individual genes. Post-initiation, RNAP II associates with negative elongation factor (NELF) exhibiting proximal promoter pause, 50–100 base pairs downstream of transcription start sites. The CDK9-Cyclin T (CycT) complex, referred to as positive Transcription Elongation Factor b (P-TEFb), is activated by CDK7-mediated phosphorylation of CDK9. The CDK9-CycT complex phosphorylates NELF and RNAPII at ser2, releasing RNAP II from promoter-proximal pause to drive productive elongation. CDK10, CDK11, and CDK12/CDK13 aid in splicing and/or termination of transcription by phosphorylating RNA cleavage and polyadenylation factors.

Cyclin-dependent kinases (CDKs) are serine/threonine kinases whose activity is regulated by binding to cyclin partners and, in many cases, by activation-loop (T-loop domain) phosphorylation. Historically, many CDKs (e.g., CDK1, CDK2, CDK4/6) have been studied largely for their roles in cell cycle transitions (G1→S, G2→M) and checkpoint regulation. However, a distinct subset of CDKs—commonly termed transcription-associated CDKs (tCDKs)—play critical roles in regulating transcription by phosphorylating RNA Polymerase II (RNAP II), modulating promoter clearance, pause-release, elongation, RNA processing, and coupling to genome-stability pathways [1,2]. Given the high transcriptional demands of malignantly transformed cells (including so-called “transcriptional addiction”), dysregulation of tCDKs has emerged as a hallmark of many cancer types [3]. As depicted in Figure 1, the family of tCDKs in human cells includes CDK7, CDK8 (and its paralog CDK19), CDK9, CDK10, CDK11, CDK12 (and its paralog CDK13), among others [1,4]. Comparative genomics suggests that tCDKs co-evolved with the extended C-terminal domain (CTD) of RNAP II heptad repeats in higher eukaryotes, enabling more complex regulation of transcription elongation, processing, and pausing control [1]. The CTD acts as a scaffold for the “mRNA factory” and contains repeats of the evolutionary conserved heptapeptide consensus sequence Tyr1-Ser2-Pro3-Thr4-Ser5-Pro6-Ser7 [5]. The CTD undergoes reversible phosphorylation by tCDKs and cycles between hypophosphorylated RNAP II, which enters the pre-initiation complex, and hyperphosphorylated RNAP II, which enables sequential transcript elongation [5]. Structural and sequence divergence among tCDKs (and their cyclin partners) underlies functional specialization and potential paralog divergence (e.g., CDK8 vs. CDK19; CDK12 vs. CDK13) [2]. However, we also note that tCDKs intersect with cell cycle-regulating CDKs (e.g., CDK7 activates cell cycle CDKs), reinforcing functional overlap. We briefly describe individual tCDKs and their biological roles in normal and cancer settings and then focus on their emerging roles in GI tumors (particularly esophageal, gastric, pancreatic, and hepatobiliary cancers), including their dysregulation in oncogenesis, therapeutic targeting, challenges, and the path ahead.

#### 1.1.1. Cyclin-Dependent Kinase 7 (CDK7)

CDK7 is a serine/threonine kinase that occupies a unique and central position at the intersection of cell cycle control and transcriptional regulation. CDK7 functions as part of a trimeric complex with cyclin H and MAT1, serving as the principal CDK-activating kinase (CAK). Through T-loop phosphorylation (i.e., phosphorylation and stabilization of the enzyme’s T-loop activation domain), CDK7 activates other CDKs, including CDK1, CDK2, CDK4, and CDK6, thereby enabling orderly cell cycle progression through G1, S, G2, and M phases [6]. Another important aspect of CDK7 is that it is an integral component of the transcription factor IIH (TFIIH) complex, where it phosphorylates serine residues in the C-terminal domain (CTD) of RNAP II. CDK7 (within the TFIIH/CAK complex) phosphorylates the RNAP II CTD at Ser5 and Ser7 of its heptad repeats, facilitating 5′ capping enzyme recruitment and promoter escape [7]. Through its dual role, CDK7 also T-loop phosphorylates other CDKs, including CDK9 and CDK12/13, positioning it as a master regulator of downstream tCDK activation [7]. In this dual capacity, CDK7 coordinates cellular proliferation with transcriptional programs necessary for cell growth and differentiation.

Beyond these functions, CDK7 also modulates the activity of key transcription factors. Notably, CDK7 phosphorylates and stabilizes the oncogenic transcription factors MYC and E2F, promoting sustained expression of genes critical for nucleotide biosynthesis, metabolism, and survival [8]. Recent work has further demonstrated that CDK7 phosphorylates the oncogenic transcriptional co-activators YAP and TAZ, protecting them from proteasomal degradation and enabling their pro-proliferative gene expression programs [9]. These observations indicate CDK7’s broader role as a regulator of transcriptional networks that support both normal and malignant cellular states that are particularly relevant in tumors characterized by transcriptional addiction. For example, small-cell lung cancer (SCLC) is characterized by transcriptional addiction, rendering tumor cells highly sensitive to CDK7 inhibition. In SCLC models, CDK7 blockade disrupts global transcription, induces DNA damage, and activates type I interferon responses, which synergize with immune checkpoint inhibitors to enhance tumor clearance [10]. Collectively, these findings highlight CDK7 as a pivotal regulator of oncogenic transcription and a promising therapeutic vulnerability across diverse malignancies. Given its dual role in cell cycle activation and transcriptional control, CDK7 represents a compelling target whose inhibition may deliver therapeutic benefit, particularly in cancers characterized by transcriptional addiction and oncogene-driven proliferation.

#### 1.1.2. Cyclin-Dependent Kinase 8 (CDK8) and Cyclin-Dependent Kinase 19 (CDK19)

The evolutionarily conserved mediator complex consists of CDK8 or its paralog CDK19, cyclin C (CCNC), mediator complex subunit 12 (MED12), and mediator complex subunit 13 (MED13) [11]. CDK19 shares approximately 91% sequence homology to CDK8, with sequence conservation in their kinase and cyclin-binding domains, while differing in their C-terminal tails [12,13]. CDK8 and CDK19 are part of a mediator kinase module that phosphorylates the CTD of RNAP II, regulating transcription or phosphorylating transcription factors to modulate their activity or promote their degradation [14]. Illustrating its essentiality, CDK8 deletion in a murine model induced embryonic lethality, most likely due to transcriptional inhibition [15]. Moreover, CDK8 deletion upregulated MYC-Threonine-58 phosphorylation and targeted it for degradation [15], indicating that CDK8 controls post-translational MYC stability. Likewise, double knockout of CDK8 and CDK19 attenuated proliferative capacity of intestinal organoids but did not induce lethality or alter differentiation [13]. First identified as a colorectal cancer oncogene, CDK8 is frequently amplified in colon carcinomas and enhances β-catenin-driven transcription, which in turn promotes proliferation; analogously, RNAi-mediated CDK8 suppression in β-catenin-activated cell lines reduces tumorigenicity [16]. In contrast, in a murine colitis-associated chemical model of intestinal tumorigenesis, CDK8 deletion did not affect tumor incidence, indicating it as dispensable for efficient tumorigenesis [13].

CDK19 expression is also upregulated in various cancers, particularly in aggressive prostate carcinoma, hepatocellular carcinoma (HCC), and breast, bladder, and gastrointestinal tumors [17,18]. Functionally, dual inhibition of CDK19 and CDK8 with small molecule inhibitors (e.g., cortistatin A), or genetic depletion, overrides TGF-β/BMP-induced epithelial-to-mesenchymal transition (EMT) in ovarian, pancreatic, and breast cancer cells, impairing tumor cell invasion and nuclear localization of the oncoprotein YAP1 [18]. Paradoxically, depending on the cellular context, CDK8 can either promote oncogenesis or act as a tumor suppressor. Its unique duality, and emerging CDK8-selective inhibitors, position CDK8 as a compelling target for precision oncology. CDK19, by contrast, acts as a transcriptional co-regulator and oncoprotein that promotes aggressive tumor phenotypes. Its selective inhibition reverses aggressive phenotypes, invasion, and castration resistance in prostate cancer models, demonstrating its potential as a therapeutic target in transcriptionally driven malignancies [17].

#### 1.1.3. Cyclin-Dependent Kinase 9 (CDK9)

Normally, RNAP II pauses at proximal promoters, and, upon additional phosphorylation signals, carries out productive elongation. Cyclin-dependent kinase 9 (CDK9) forms a complex with cyclin T1 and cyclin T2, together referred to as transcription elongation factor b (P-TEFb). P-TEFb phosphorylates Ser2 in the CTD of RNAP II, facilitating promoter-proximal pause-release and transcription elongation of most mRNAs [11]. Several studies have shown direct interaction of P-TEFb with transcriptional activators such as MYC and BRD4, facilitating their recruitment to transcriptional machinery at promoter regions [19]. In cancer cells, MYC accumulates in the promoter regions of its target genes and recruits P-TEFb to promote RNAP II-mediated transcription. Such tumors rely on MYC for sustained proliferation, and CDK9 inhibition hampers MYC-driven tumor growth by abrogating MYC-induced oncogenic pathways and downstream anti-apoptotic proteins such as MCL1 [20].

In solid tumors, CDK9 targeting has demonstrated therapeutic promise. For example, in glioblastoma multiforme (GBM), CDK9 inhibition reduces MCL1, disrupts mitochondrial function, and suppresses tumor growth in xenograft models [21]. Endometrial cancer cells also exhibit dependence on CDK9; siRNA knockdown or selective inhibitors (e.g., LDC067) decrease RNAP II phosphorylation, reduce MCL1, and induce apoptosis [20]. CDK9 is a central transcriptional kinase whose pharmacological inhibition disrupts key survival pathways in both hematologic and solid tumors. With compounds ranging from broad (flavopiridol, dinaciclib) to highly selective (atuveciclib, VIP152, KB-0742, LDC067, and more) CDK9 inhibitors, current developments such as proximity-based approaches (e.g., molecular glues) focus on maximizing anti-cancer activity by increasing specificity while reducing toxicity profiles. Future success will likely depend on combination strategies to overcome adaptive resistance and translate CDK9 targeting into clinical benefit.

#### 1.1.4. Cyclin-Dependent Kinase 10 (CDK10)

CDK10 primarily binds cyclin M (also known as cyclin Q), which protects CDK10 from proteasomal degradation. CDK10 is involved in transcription elongation and splicing and phosphorylates ETS2 to inhibit its transactivation by promoting proteasomal degradation [22]. In a mouse model, genetic deletion of CDK10 showed growth retardation and skeletal abnormalities [11], while in a Swedish breast cancer cohort, the *CDK10* gene was deleted in 80% of cases [23]. In lung adenocarcinoma, compared to adjacent normal tissues, decreased CDK10 expression was associated with distant metastasis, higher TNM stage, and shortened overall survival. In lung cancer cells, CDK10 binds and inactivates ETS2, thereby suppressing c-RAF/p-MEK/p-ERK signaling, decreasing expression of matrix metalloproteinase, and inhibiting cell invasion and metastasis [22]. Furthermore, depletion of CDK10 enhanced cancer cell proliferation, metastasis, and radioresistance via activation of the JNK/c-JUN pathway [24]. CDK10 was also identified as a downstream target of RING Finger Protein 115-driven tumorigenesis, and overexpression of CDK10 neutralized malignant phenotypes in a thyroid carcinoma model [25]. Likewise, CDK10 knockdown promoted glioma metastasis via zinc finger protein SNAI1 (Snail), while CDK10 overexpression inhibited glioma cell proliferation and metastasis [26]. In contrast, the *CDK10* gene is upregulated in colorectal and prostate cancer. In colorectal cancer, CDK10 overexpression increased cell proliferation and decreased responsiveness of cancer cells towards chemotherapeutic drugs [27]. In conclusion, more studies are needed to dissect the tumor-promoting or -suppressive roles of CDK10 in different cancers, and their underlying mechanisms.

#### 1.1.5. Cyclin-Dependent Kinase 11 (CDK11)

Two distinct human genes, *CDK11A* and *CDK11B*, encode CDK11 isoforms, and the longer 110 kDa isoform (CDK11^p110^) is ubiquitously expressed in cells, while the shorter 58 kDa isoform is expressed exclusively in G2/M phase cells [11,28]. CDK11 binds the functionally redundant cyclinL1 or cyclinL2 to mediate transcription and splicing, and is elevated in breast cancer and associated with poor clinical outcomes [11,28]. Specifically, CDK11 phosphorylates SF3B1, a core component of the spliceosome complex, and activates pre-mRNA splicing, and a CDK11 oral inhibitor, OTS964, blocked hyperphosphorylation of SF3B1 to inhibit spliceosome activation [28]. In terms of transcriptional control, CDK11 regulates RNAP II at a checkpoint upstream of CDK9, and inhibiting CDK11 results in acute loss of RNA synthesis [29]. CDK11 inhibition decreased BCL2 levels and the induction of autophagy in a breast cancer model, decreasing proliferation and migration [30]. CDK11 is essential for survival and proliferation of aggressive acute myeloid leukemia (AML) cells, and its inhibition rapidly decreased hematological tumor burden in a mouse AML model [29]. CDK11 also promoted CRL4-mediated ubiquitination and degradation of Large Tumor Suppressor Kinase 1 (LATS1). Moreover, a CDK11/cyclinL2 complex phosphorylated NF2, reducing its binding with CRL4 to inactivate the Hippo pathway in cervical cancer cells [31]. CDK11 knockdown also inhibited ovarian cancer cell growth in vivo and in vitro and enhanced the cytotoxicity of paclitaxel [32]. Given the involvement of CDK11 in different cellular functions, careful delineation of its tumor-specific dependencies will be important.

#### 1.1.6. Cyclin-Dependent Kinase 12 (CDK12) and Cyclin-Dependent Kinase 13 (CDK13)

CDK12 and CDK13 form distinct complexes with cyclin K and carry out fundamental functions in transcriptional regulation. CDK12 and CDK13 have 43% sequence homology and have similar kinase domains with a conserved PITAIRE motif. CDK12, CDK13, and cyclin K are required for transcriptional gene elongation and cell viability, as their knockout has embryonic lethality [33]. Specifically, a cyclin K-CDK12 complex phosphorylates the RNAP II CTD at ser2 to regulate DNA damage response genes, and loss of cyclin K and CDK12 sensitizes cells to DNA-damaging agents [33]. CDK12 is reported as a driver gene inducing liver metastasis in colorectal cancer (CRC), and its inhibition by shRNA or a selective inhibitor (SR-4835) suppressed proliferation, survival, stemness, and transcription of SE-associated oncogenes [34]. Experimental overexpression of CDK12 in murine mammary epithelium is sufficient to initiate breast tumorigenesis and metabolic reprogramming, particularly activation of serine-glycine-one carbon (SGOC) metabolism, causing sensitivity to methotrexate-based therapies in xenograft and clinical cohorts [35]. In a HER2-positive breast cancer model that developed resistance to lapatinib via activation of PI3K/AKT signaling, blockade of CDK12 resensitized tumors to anti-HER2 treatment [36]. In metastatic castration-resistant prostate cancer (mCRPC), biallelic loss or mutation of CDK12 correlates with genomic tandem duplications, elevated neoantigen load, immune infiltration, and responsiveness to immune checkpoint blockade [37]. Although a CDK12/CDK13 dual inhibitor, ZSQ836, exerted potent antitumor effects, it contrastingly hampered T-cell infiltration and activation in ovarian cancer-bearing mice [38]. Inhibition of CDK12 was also sufficient to confer sensitivity towards PARP inhibitors in breast cancer and ovarian cancer models [39].

In contrast to its oncogenic functions, recurrent CDK12 inactivation was also reported in tubo-ovarian high-grade serous carcinoma (HGSC), while it was also shown to be a bona fide tumor suppressor in prostate cancer [40,41]. Ablation of CDK12 increased aggressiveness and DNA damage in a triple knockout transgenic mouse model, where tumor-suppressors *Trp53*, *Rb1*, and *Nf1* were inactivated [40]. Surprisingly, targeting CDK13 with an orally bioavailable degrader of CDK12/CDK13, YJ1206, in CDK12-null cells sensitized them to therapy and alleviated aggressiveness of CDK12-null tumors [40]. Thus, CDK12 acts either as a safeguard of genome integrity or as an oncogenic driver. Altering this kinase offers diagnostic, prognostic, and therapeutic potential through combination therapies such as pairing CDK12 inhibition with PARP inhibitors, immunotherapies, or targeted agents, in HER2-overexpressing tumors.

CDK13 is significantly upregulated in prostate tumors, and its knockdown results in reduced tumor cell proliferation and total lipid content, showcasing its potential metabolic vulnerability [42]. Recent phosphoproteomic studies demonstrate that CDK13 interacts with and phosphorylates NSUN5 Ser-327 in prostate cancer, leading to increased acetyl-CoA carboxylase (ACC1) mRNA stability, enhancing fatty acid synthesis and lipid accumulation to promote tumor growth [42]. Further, CDK13 is required for RNA surveillance, and CDK13 mutation causes aberrant RNA stabilization that leads to abnormal protein-coding transcript translation, promoting melanoma in zebrafish [43]. Together, these functions make CDK13 a promising therapeutic target in metabolically driven cancers, and selective inhibitors could modulate transcription, RNA processing, and metabolic flux for antitumor efficacy.

## 2. tCDKs in GI Malignancies

### 2.1. Esophageal Cancer

Although cell cycle-dependent tCDKs are implicated in cancer stem cell (CSC) functionality, the mechanistic role of tCDKs in regulating CSC properties remains largely unknown [44]. Either CDK7 knockdown or covalent CDK7 inhibitor (THZ1) treatment of KYSE410 esophageal squamous cell carcinoma (ESCC) cells reduced spheroid-formation capacity, a marker of stemness, and reduced transcripts and protein expression of stemness-associated genes such as SOX9, SOX2, OCT4, and NANOG [45]. Moreover, genetic CDK7 depletion reduced tumor growth, while THZ1 treatment potentiated chemotherapy (cisplatin) benefit in a KYSE410 xenograft model [45]. RNA sequencing revealed that low-dose THZ1 treatment in an ESCC model depleted super-enhancer (SE)-associated novel oncogenes such as PAK4, RUNX1, DNAJB1, SREBF2, and YAP1, indicating these oncogenic transcripts to be regulated by CDK7 in ESCC [45]. Indeed, CDK7 interacted with YAP1 in KYSE410 cells, caused YAP phosphorylation at S127 and S397, co-localized with nuclear phosphorylated YAP1, and regulated YAP1 activity [44]. The dual CDK7/9 inhibitor, SNS-032, successfully inhibited cellular viability, retarded anchorage-independent growth, and potentiated ESCC cell sensitivity to cisplatin in vitro and in vivo [46]. Moreover, SNS-032 induced mitochondrial-dependent apoptosis of ESCC cells by reducing Mcl-1 transcription, and potently abrogated ESCC cell migration and invasion potential through transcriptional downregulation of MMP-1 [46]. Notably, SNS-032 inhibited the growth of ESCC xenografts, increased overall survival, and reduced lung and lymph node metastasis in nude mice [46].

LINC00094 is an SE-associated, competing endogenous long noncoding RNA (ce-lncRNA) that promotes ESCC cell growth via TCF3 and KLF5 transcription factor binding to SE regions. CDK7 inhibitor THZ1 treatment inhibited binding of TCF3 and KLF5 to SE regions, and attenuated expression of LINC00094, in ESCC cells [47]. Likewise, C-terminal-binding protein 2 (CtBP2) transcriptionally repressed epithelial-specific genes such as E-cadherin, while CtBP2, CDK7, and cyclin H expression were also higher in ESCC tissues with lymph node metastasis, versus those without lymph node metastasis. CtBP2 also physically interacted with CDK7/Cyclin H to promote the epithelial–mesenchymal transition (EMT) in ESCC cells [48].

CSCs differ from tumor cells in terms of metabolic features such that tumor cells rely on glycolysis, while CSCs depend on oxidative phosphorylation; interestingly, such CSC metabolic dependency was regulated by CDK7 in ESCC where a CDK7-YAP axis increased lactate dehydrogenase D (LDHD) expression, catabolizing D-lactate to pyruvate [44]. CDK7 overexpression also elevated pyruvate levels while decreasing D-lactate, and pyruvate treatment promoted sphere-formation capacity, and stemness-associated gene expression, in a dose-dependent manner, thereby regulating tumorigenic potential and maintenance of ESCC-CSC stemness [44]. In contrast to CDK7 promoting cancer stemness, CDK8 expression is associated with longer overall survival in ESCC [49].

As a component of the ubiquitin-ligase complex, F-box protein 32 (FBXO32) expression positively correlates with better overall survival in esophageal cancer (EC) patients and suppresses EC cell proliferation and metastasis [50]. Mechanistically, FBXO32 physically interacts [50] with CDK9, leading to CDK9 degradation via ubiquitination [51]. BAY1143572, a CDK9 inhibitor, showed synergistic effects with 5-flurouracil and inhibited esophageal adenocarcinoma (EAC) cell proliferation in vitro and in xenografts [52]. CDK9 inhibition also decreased MCL1 expression via suppressing HIF1α binding to the MCL1 promoter [52]. Moreover, BAY1143572 synergistically increased radiosensitivity in an EAC model [53]. Expression of the long isoform of CDK11 (CDK11^p110^) was also significantly elevated in ESCC compared to normal tissues, while RNA interference-mediated abrogation of CDK11^p110^ suppressed proliferation (arrested cells in G2/M phase), clonogenicity, and migratory potential [54]. Analysis of online databases indicated increased CDK12 expression in ESCC tissues, as associated with poor overall survival and prognosis, while CDK12 knockdown in KYSE150 cells reduced proliferation [55]. H63, a novel CDK12 inhibitor from a series of 4-(2-(methylamino) thiazol-5-yl) pyrimidin-2-amine derivatives, exhibited anti-tumorigenic efficacy in esophageal cancer in vitro and in vivo. H63 also inhibited phosphorylation of RNAP II CTD Ser2, abrogating transcription elongation and eliciting G1 phase cell cycle arrest and cell apoptosis. Moreover, H63 induced DNA damage through a CDK12-ATM/ATR-CHEK1/CHEK2 signaling axis [55].

### 2.2. Gastric Cancer

Immunohistochemistry analysis of 173 gastric cancer (GC) tissues indicated that CDK7 was significantly increased in tumor tissues and positively associated with tumor grade and lymph node metastatic infiltration [56]. Furthermore, CDK7 expression correlated with Ki-67 proliferation marker staining and indicated poor GC prognosis [56]. Analogously, in GC cells, ectopic expression of CDK7 led to increased proliferation, while its knockdown reduced cell proliferation [56]. Pharmacologically, treatment with BS-181, a selective CDK7 inhibitor, decreased cell proliferation, migration, and invasion in GC cell lines. Mechanistically, BS-181 induced apoptosis by BCL-2 downregulation, while the pro-apoptosis effectors Bax and caspase-3 were significantly elevated [56]. Another selective and irreversible CDK7 inhibitor, THZ2, abrogated GC cell growth, induced G2/M cell cycle arrest, and led to apoptosis by increasing intracellular reactive oxygen species [57].

Regarding CDK8, this kinase was downregulated by miR-26b-5p in GC cells, reducing cell proliferation through inhibitory effects on STAT3 signaling. Analogously, CDK8 overexpression increased p-STAT3 and activated the STAT-3 pathway, leading to cell growth [58]. Immunohistochemistry analysis across 12 adenomas, 24 early gastric carcinomas, 24 advanced gastric carcinomas, and 21 metastatic lymph nodes demonstrated a positive correlation between CDK8 expression, tumor growth, and lymph node metastasis [59]. Furthermore, CDK8 expression positively associated with β-catenin activation in gastric adenocarcinoma tissues [59], and RNAi of CDK8 suppressed β-catenin expression in gastric cancer cells [59]. CDK8 was also reported to be a target gene of miR-107, and a miR-107 inhibitor decreased CDK8 mRNA and protein levels in GC cell lines [60].

In contrast to CDK8, mRNA and protein expression of CDK10 was significantly reduced in GC tissues versus matched adjacent normal tissues, and decreased CDK10 expression was associated with poor survival in GC patients [61]. CDK10 suppression in gastric cancer is associated with chromosome 16 q24 deletion. However, further research is warranted to assess CDK10 suppression via promoter hypermethylation or loss of heterozygosity at the q24 region of chromosome 16 [61].

**Figure 2:**
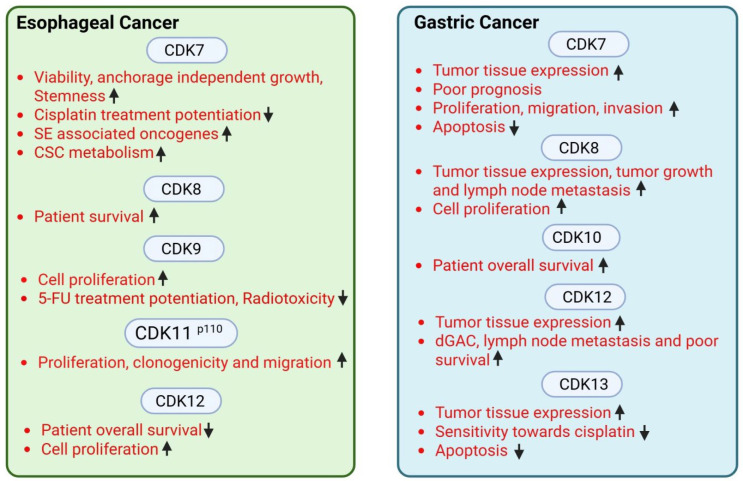
Expression, cancer-associated functions and clinical relevance of tCDKs in esophageal and gastric cancers. An upward arrow indicates increase and a downward arrow indicates decrease (www.BioRender.com).

CDK12 was also discovered as a metastasis marker gene by single-cell RNA sequencing of primary GC tumor tissues and paired lymph node metastatic tissues [62]. Functionally, CDK12 induced GC cell proliferation, migration, and angiogenesis through activation of PI3K/AKT/mTOR signaling [63]. Another study reported a similar trend, with high CDK12 expression observed in GC tissues significantly correlated with diffuse type gastric adenocarcinoma (aggressive phenotype), lymph node metastasis, and poor overall survival [64]. Additionally, CDK12 expression showed positive correlation with CD8^+^ cells and CCL21 mRNA [64], while another study showed decreased CDK12 protein expression in GC tissues compared to adjacent non-tumor tissues [65]. However, another report showed that low CDK12 levels correlated with advanced stage tumors, poor differentiation, and worse survival [65]. Expression of CDK13 circular RNA (circ-CDK13), derived from the CDK13 transcript, was increased in GC clinical samples, while its overexpression attenuated sensitivity and apoptosis of GC cell lines towards cisplatin. Further, circ-CDK13 levels were downregulated by methionine restriction in cisplatin-resistant GC cells [66]. CDK13 was also shown to be a direct target of HMGA2, and co-targeting HMGA2 and CDK13 effectively reduced GC cell growth [67]. Figure 2 summarizes tCDK functions in esophageal and gastric cancers.

### 2.3. Pancreatic Cancer

The KPC mouse model (LSL-^KrasG12D/+^;LSL-Trp53^R172H/+^;Pdx-1-*Cre*) is the most commonly used genetic model for pancreatic ductal adenocarcinoma (PDAC). A KPC model-derived cell line, TB32047, was used to screen CRISPR-Cas9-based small guide RNAs, indicating that CDK7 is a kinase involved in mediating chemoresistance to paclitaxel and gemcitabine [68]. Either CDK7 gene knockout or its pharmacological inhibition by THZ1 induced cell cycle arrest, apoptosis, and DNA damage through downregulation of a STAT3-MCL1-CHK1 axis. Moreover, CDK7 inhibition potentiated gemcitabine-paclitaxel co-treatment in cell lines, as well as reduced tumor growth [68]. Since PDAC cells are addicted to CDK7-mediated transcription, CDK7 inhibition by THZ1 reduced serine phosphorylation of the RNAP II CTD and decreased cell viability and abrogated PDAC tumor progression [69]. This study also reported that MYC expression was necessary to promote CDK7 inhibitor-mediated PDAC cell killing, and PDAC cells resistant to THZ1 showed decreased MYC protein expression [69]. High CDK7 expression was also associated with worse OS and disease-free survival. Further, a CDK7 inhibitor more robustly suppressed KRAS^G12V^ PDAC cell viability compared to cells with KRAS^G12D^ mutations; however, another covalent CDK7 inhibitor, YKL-5-124, did not show KRAS mutation selectivity [70]. ChIP assays also identified a greater number of H3K27-acetylation-bound super-enhancer (SE) regions and H3K27ac enrichment in SE-related genes in PDAC cells with KRAS^G12V^ compared to KRAS^G12D^ mutations; analogously, CDK7 inhibition abrogated SE region activity in KRAS^G12V^-mutated cells [70]. Mechanistically, THZ1 inhibited binding of H3K27-acetylated histones to PIK3CA and lowered phosphorylation of AKT and mTOR to inhibit their downstream effectors [70].

SE landscapes, profiled by H3K27-acetylation marks, are linked with robust and constitutive transcription of oncogenes, and CDK7 and BRD4 are key components of SE complexes that positively regulate SE-driven transcription [71]. Consequently, combining CDK7 and BRD4 inhibitors in a nanoparticle formulation strongly induced cell cycle arrest and abrogated tumor growth of gemcitabine-refractory PDAC patient-derived xenografts [71]. Higher CDK7 activity also correlated with unfavorable prognosis in a mass-spectrometry-based phospho-proteomics study of 42 resected PDAC tissues [72]. Another study explored the vulnerabilities of mutant KRAS (mtKRAS)-dependent cancer by screening druggable proteins by global phosphor-proteomic data analysis in a panel of mtKRAS-dependent or -independent cancer cell lines [73]. AT7519, a CDK 1, 2, 7, and 9 inhibitor, selectively killed mtKRAS-dependent PDAC cells [73], and AT7519 treatment blocked phosphorylation of CDK7 (RNAP II; Ser5) and CDK9 (RNAP II; Ser2) substrates to induce apoptosis in 2D and 3D mtKRAS PDAC cells. AT7519 also suppressed tumor growth of patient-derived xenografts [73].

Regarding CDK8, in a KRAS^G12D^ -driven pancreatic adenocarcinoma (PDAC) model, CDK8 promoted resistance to the small molecule inhibitor MRTX1133 in KRAS^G12D^ cells. CDK8 also remodeled the tumor microenvironment and promoted immune evasion by CXCL12 secretion and inhibiting Fas expression. Correspondingly, targeting CDK8 alone or with αCTLA-4 immunotherapy overcame resistance to a KRAS^G12D^ inhibitor, prolonging survival [74]. CDK8 expression was also higher in pancreatic cancer tissues with mtKRAS as compared to wild-type KRAS (WT-KRAS) [75]. mtKRAS abrogation downregulated CDK8 expression, while ectopic expression of mtKRAS in pancreatic cancer cells with WT-KRAS increased CDK8 expression, possibly through HIF1α [75]. Further, CDK8 induced cell proliferation, inhibited apoptosis, and promoted cell migration and invasion in pancreatic cancer cells via a Wnt/β-catenin signaling pathway [75]. Another study established a histology-based addiction of PDAC towards transcriptional CDK inhibition [76].

Predominantly, PDAC is classified as “well differentiated”, as a classical phenotype, and as “poorly differentiated” or “basal phenotype” with poor overall prognosis [76]. Therapeutic vulnerability of basal type PDAC cell lines towards THZ-induced CDK7 inhibition was attributed to the absence of the histone deacetylase SIRT6, resulting in failure to activate a master regulator of stress response, activating transcription factor-4 (ATF-4), making basal cells more sensitive to tCDK inhibition [76]. Furthermore, chemical or genetic abrogation of CDK9 also showed therapeutic vulnerability in basal compared to classical PDAC cell lines [76]. Pancreatic cancer spheroid cultures showed cancer stem cell (CSC)-like properties, with increased resistance to gemcitabine treatment and activation of NOTCH signaling; analogously, injection of spheroidal cultures into immunocompromised mice showed larger tumors than tumors formed by 2D culture. In this model, CDK7 inhibition impacted growth of spheroids, indicating that CDK7 is associated with CSC survival and functionality [77]. Likewise, Wei et al., explored the role of CDK8 in angiogenesis in a pancreatic cancer model, revealing increased nuclear staining of CDK8 in pancreatic cancer tissue specimens compared to noncancerous adjacent tissues. Further, CDK8 overexpression induced angiogenesis through a CDK8-β-catenin-KLF2 axis [78].

Similar to CDK8, CDK9 is overexpressed in pancreatic cancer tissues, compared to normal tissues, and is even elevated in well-differentiated pancreatic cancer. Specifically, high CDK9 expression is associated with shortened survival and poor prognosis, while a CDK9 inhibitor, SNS-032, reduced pancreatic cell viability and survival by inducing cell cycle arrest and apoptosis [79]. Another approach, aminopyrazole-based proteolysis-targeting chimeras (PROTACs), selectively degraded CDK9 and showed synergistic cell growth inhibition with the Bcl-2 inhibitor venetoclax in MiPaCa2 cells [80]. The Polycomb proteins su(var)3-9, enhancer of zeste, and trithorax (SET) localize to oncogene transcriptional start sites and promote oncogenic RNA transcription, yielding a transcriptomic profile similar to that of CDK9 overexpression in PDAC cells [81]. SET blocked activity of PP2A, a validated phosphatase that dephosphorylates the RNAP II CTD and suppresses global transcription [81]. A novel CDK9 inhibitor, Atuveciclib, in combination with tumor necrosis factor-related apoptosis-inducing ligand (TRAIL), induced cell death and suppressed the anti-apoptosis protein MCL1 in PDAC cells, as well as in gemcitabine-resistant and patient-derived xenograft cell lines [82,83,84]. Another novel 2,4-disubstituted pyrimidine derivative showed selectivity toward CDK9, decreasing RNAP II ser2 phosphorylation, inducing apoptosis and cell cycle arrest to reduce PANC-1 cell xenograft progression [85]. UNC10112785, another potent CDK9 inhibitor, repressed Myc protein at the transcriptional and post-transcriptional levels to suppress mtKRAS PDAC growth in 2D and 3D cultures [86]. CDK9 or CDK12 inhibition also antagonized aberrantly activated Hedgehog (Hh) signaling and targeted cells resistant to FDA-approved Hh-targeting smoothened inhibitors (SMOis) [87]. Beta-1.4-galactosyltransferase 1 (B4GALT1) mediated N-linked glycosylation of CDK11^p110^ and increased cancer progression and chemoresistance to gemcitabine in a PDAC model [88]. Increased expression of CDK12 was also correlated with poor prognosis in PDAC [89].

### 2.4. Hepatobiliary Cancer

In liver cancer, CDK7 physically interacts with β-catenin to promote binding of β-catenin binding to TCF4, enhancing growth and migration of hepatocellular carcinoma (HCC) cells [90]. HCC cell lines were also found to be susceptible to the CDK7 inhibitor THZ1, which also reduced HepG2 tumor xenograft growth [91]. Specifically, THZ1 caused cell cycle arrest and DNA damage-induced cell death, and reduced MYC oncoprotein expression. Further, overexpression of MYC sensitized HCC cells towards THZ1 treatment [92]. Furthermore, MYC-driven liver tumors showed dependency on CDK9-mediated elongation, whereas abrogation of CDK9 by shRNA elicited a robust, anti-tumorigenic response [93]. In super-enhancer (SE) complexes, well implicated in liver tumorigenesis driving continuous oncogene transcription [94], CDK7 is a SE complex component that is frequently overexpressed in HCC tissues. HCC cells were also susceptible towards CRISPR/Cas9 mediated perturbations of CDK7, along with other factors of SE complexes such as bromodomain-containing protein 4 (BRD4), E1A-binding protein P300 (EP300), and mediator complex subunit 1 (MED1) [94,95]. The CDK7 inhibitor THZ1 also exerted strong anticancer effects in in vivo and in vitro HCC models, suggesting that SE complex targeting is a promising therapeutic strategy [94]. Moreover, silencing of the long non-coding RNA HEIH, which directly interacts with microRNA (miR)-193a-5p and CDK8, reduced proliferation, migration, invasion, and in vivo tumor progression in an HCC model. Analogously, overexpression of CDK8 restored the anti-tumorigenic effects of HEIH silencing, indicating CDK8 as an effector of HEIH [96].

**Figure 3:**
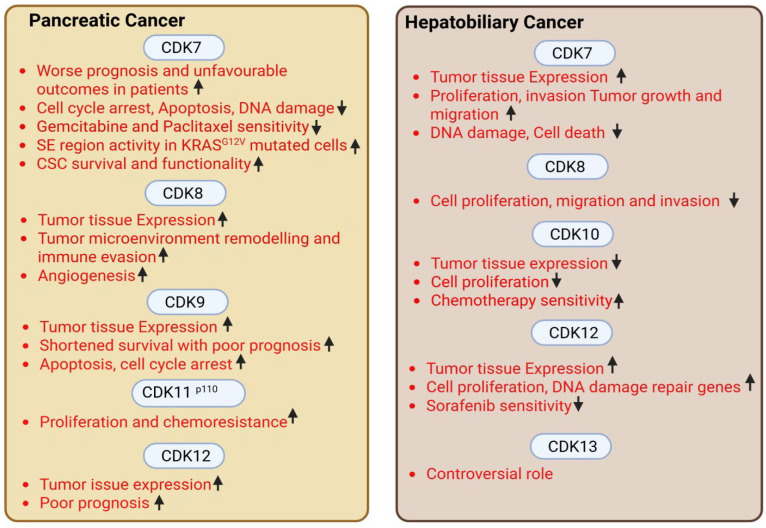
Expression, cancer-associated functions and clinical relevance of tCDKs in pancreatic and hepatobiliary cancers. An upward arrow indicates increase and a downward arrow indicates decrease (www.BioRender.com).

Conversely, CDK10 was reported as a tumor suppressor in hepatobiliary cancers, with ectopic expression of CDK10 decreasing cell proliferation in HCC cell lines (decreasing cells in S-phase); also, CDK10 mRNA and protein expression were reduced in HCC tumors compared to normal liver tissues [27]. Additionally, overexpression of CDK10 increased sensitivity of HCC cells, while CDK10 knockdown decreased sensitivity of biliary tract cancer cells towards chemotherapy. In ERBB2-amplified biliary track cancer, CDK12 alterations were found as novel targets [97]. A non-biased CRISPR screen identified CDK12 to be critically required for most HCC cell lines, and CDK12 inhibition with shRNA or a small molecule (THZ531) resulted in robust anti-proliferative effects. THZ531 treatment also reduced expression of DNA damage repair-related genes and elicited DNA damage response in HCC cell lines. Furthermore, THZ531 combination treatment with sorafenib showed synergy and induced apoptosis in HCC cells [98]. CDK13 was reported to be a target of micro(mi)RNA-215, and inhibition of miRNA-215 upregulated CDK13 and retarded HCC development [99]. In contrast, CDK13 gene copy number and oncogenic activity were elevated and significantly associated with clinical onset age in HCC patients [100]. CDK13 RNA showed over-editing sites in HCC tissues and was associated with poor HCC prognosis [101]. Figure 3 summarizes tCDK functions in pancreatic and hepatobiliary cancers.

## 3. Key Challenges and Future Directions

Transcriptional CDKs (tCDKs) play pivotal roles in essential biological processes, including RNAP II activation, productive transcriptional elongation, RNA processing, and splicing, crucial for cancer cell growth and survival. Often cancer cells exhibit heightened dependence upon transcriptional machinery compared with normal tissues, and selectively targeting tCDKs in GI malignancies has shown dynamic effects on tumor cell growth, stemness, metastasis, and drug response in preclinical models. Despite promising preclinical anti-cancer activity, tCDK inhibitors face several challenges, such as lack of specificity, intolerant toxicities, overlapping biological functions, and the emergence of resistance [11,102,103]. Development of specific tCDK inhibitors poses a challenge due to the high level of homology among CDKs and their overlapping biological roles, potentially leading to unintended and toxic consequences for normal tissues [11]. For example, the first generation CDK inhibitor, flavopiridol, interferes with cell cycle CDK and tCDK activities and became the first CDK inhibitor to enter human clinical trials after showcasing successful anti-cancer effects in preclinical models. However, due to lack of specificity, it was associated with severe toxicities and failed to achieve desired responses [11]. Other pan-CDK inhibitors that could not show encouraging anti-tumor effects include roscovitine, SNS-032, and AZD5438. Table 1 indicates inhibitors in clinical trials targeting specific tCDKs.

Another limitation towards tCDK inhibitor usage is the emergence of resistance. For example, continuous exposure of prostate cancer cells to samuraciclib (a CDK7 non-covalent inhibitor) led to a mutation of a single base (Asp97 to Asn (D97N)) in the CDK7 gene that changed the affinity of mutant cells towards not only samuraciclib but also other non-covalent CDK7 inhibitors, causing outgrowth of mutant cells [103]. Emerging therapeutic strategies such as proteolysis-targeting chimeras (PROTACs) and molecular glues take advantage of a targeted protein degradation approach and offer ways to destroy specific oncogenic tCDK-cyclin complexes, potentially decreasing off-target effects [104]. For instance, the CDK9 PROTAC (KI-CDK9d-32) selectively degraded CDK9 and inhibited a compensatory increase in MYC protein expression (a cause of resistance towards CDK9 inhibitors), abrogating MYC-driven oncogenic programs, as compared to a conventional CDK9 inhibitor, KB-0742 [105]. Another novel therapeutic approach, regulated induced proximity targeting chimera (RIPTAC), is a heterobifunctional small molecule that links CDK inhibitors with ligand-targeting tumor-specific protein, enabling formation of intracellular ternary complexes that induce anti-proliferative effects in cancer cells [106].

**Table 1:** Clinical trials targeting tCDKs in cancer. Chemical structures were created using https://www.moldraw.com/.

Trial Identifier	tCDK Targeted	tCDK-Inhibitor Structure	Tumors Targeted
NCT04726332 [107]	CDK7	XL102 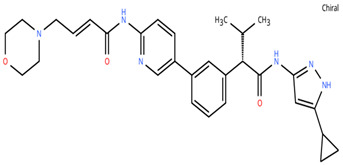	Neoplasm malignant, epithelial ovarian cancer, triple negative breast cancer hormone receptor positive (HR+) breast carcinoma, metastatic castration-resistant prostate cancer (CRPC) GI tumors: Not applicable
NCT05394103	CDK7	Q901 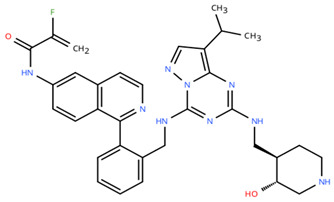	Advanced or metastatic ovarian, CRPC, HR+ HER2- breast, endometrial cancer, small-cell lung cancer GI tumors: Colorectal, Pancreatic Cancer
NCT04247126	CDK7	SY5609 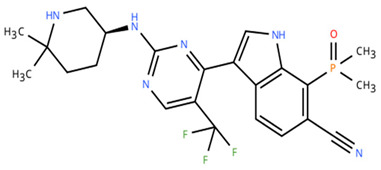	HR-positive, HER2-negative breast cancer GI tumors: Pancreatic ductal adenocarcinoma (PDAC)
NCT03134638	CDK7	SY-1365 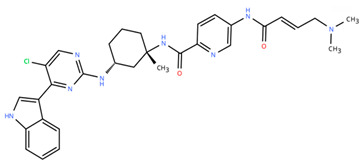	HR+ metastatic breast cancer, ovarian cancer, advanced solid tumors of any histology GI tumors: Not applicable
NCT03363893 [108]	CDK7	CT7001/Samuraciclib 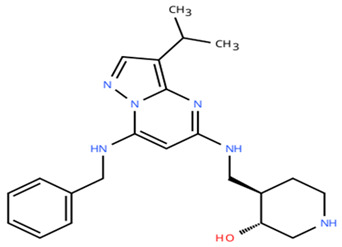	Triple negative breast cancer (TNBC), CRPC, (HER+ve)/human epidermal growth factor-2 negative (HER2-ve) breast cancer GI tumors: Not applicable
NCT03065010	CDK8/ CDK19	BCD-115 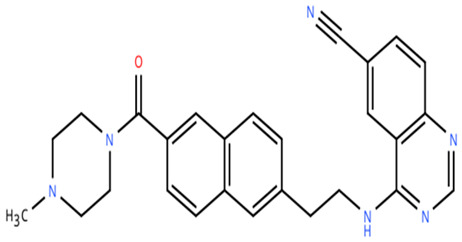	ER(+) HER2(-) local advanced and metastatic breast cancer GI tumors: Not applicable
NCT06987058	CDK8/ CDK19	RVU120/SEL120 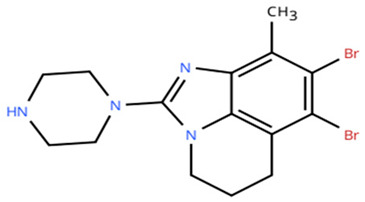	Advanced solid tumors, acute myeloid leukemia (AML), High-risk myelodysplastic syndrome GI tumors: Not applicable
NCT06268574	CDK8/ CDK19	RVU120/SEL120 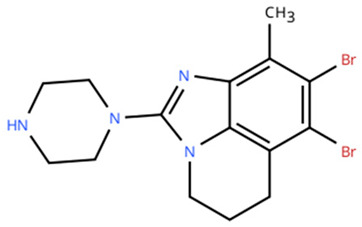	Acute myeloid leukemia (AML), High-risk myelodysplastic syndrome (MDS) GI tumors: Not applicable
NCT06243458	CDK8/ CDK19	RVU120/SEL120 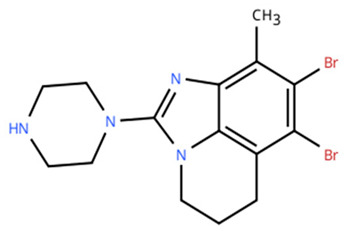	Low-risk myelodysplastic syndrome GI tumors: Not applicable
NCT06191263	CDK8/ CDK19	RVU120/SEL120 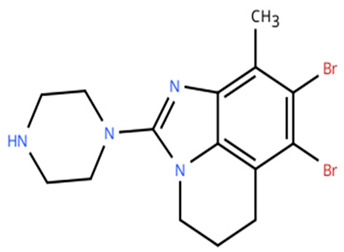	Relapsed/refractory AML GI tumors: Not applicable
NCT06532058	CDK9	QHRD107 Not available	Relapsed/refractory acute myeloid leukemia GI tumors: Not applicable
NCT04588922	CDK9	SLS009 (formerly GFH009) 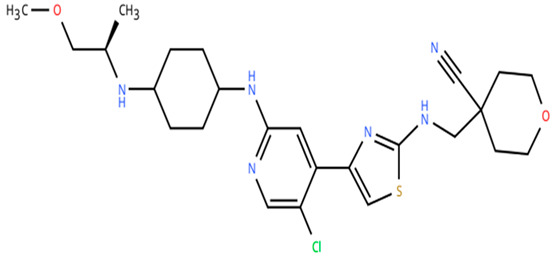	Hematologic malignancies GI tumors: Not applicable
NCT00835419	CDK9	P276-00/Riviciclib 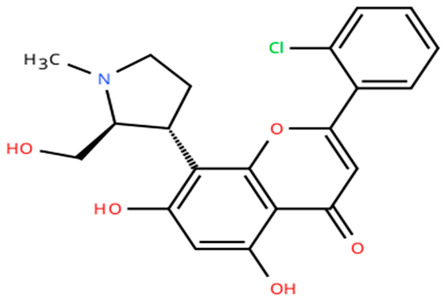	Advanced-metastatic melanoma GI tumors: Not applicable
NCT00408018	CDK9	P276-00/Riviciclib 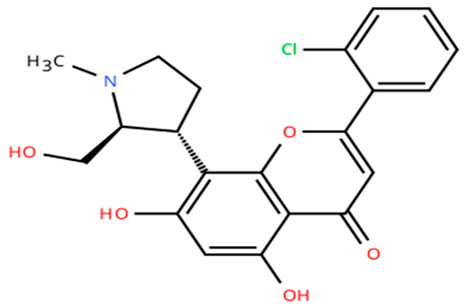	Metastatic or unresectable malignancy GI tumors: Not applicable
NCT05168904	CDK9	Fadraciclib (Formerly CYC065) 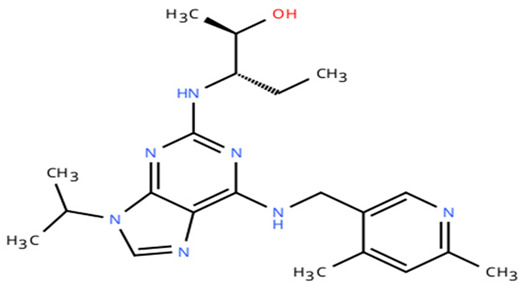	Leukemia, MDS GI tumors: Not applicable
NCT04983810	CDK9	Fadraciclib (Formerly CYC065) 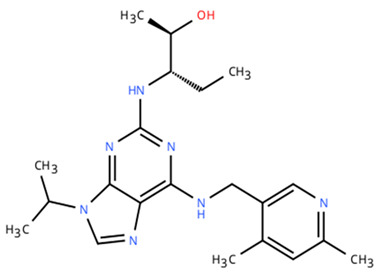	Advanced solid tumors or lymphoma, endometrial or ovarian cancer, breast cancer GI tumors: Biliary tract cancer, HCC, colorectal cancer
NCT04978779	CDK9	VIP152 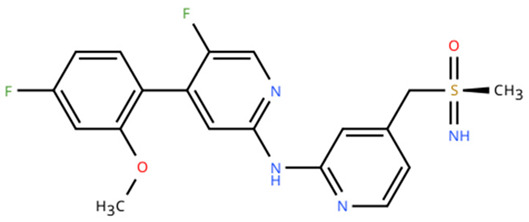	High-risk chronic lymphocytic leukemia GI tumors: Not applicable
NCT03263637 [109]	CDK9	AZD4573 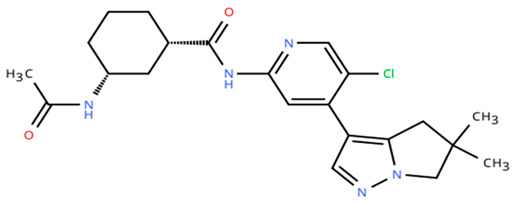	Leukemia, lymphoma GI tumors: Not applicable
NCT02745743	CDK9-	BAY1251152 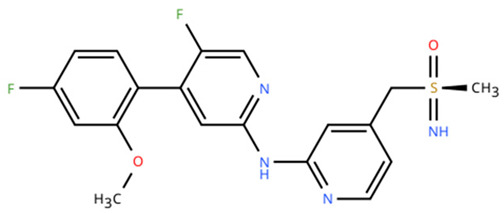	Hematological malignancy GI tumors: Not applicable
NCT04718675	CDK9	KB-0742 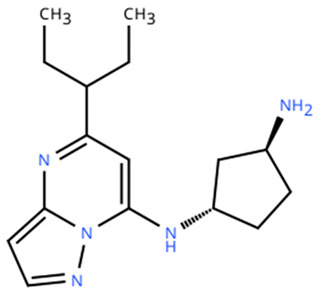	High grade serous ovarian cancer GI tumors: Not applicable
NCT05159518	CDK9	PRT2527 Not available	Advanced/metastatic sarcomas displaying documented gene fusion, castrate resistant prostate cancer, hormone receptor positive HER2-negative breast cancer, advanced/metastatic non-small cell lung cancer, and solid tumors displaying MYC amplification GI tumors: Not applicable

## 4. Conclusions

In conclusion, tCDKs are upregulated in esophageal, gastric, pancreatic, and hepatobiliary cancers, and tCDK expression was correlated with aggressiveness in GI cancers (some exceptions are listed in Figure 2 and Figure 3). While tCDK targeting offers a robust strategy for cancer care, its therapeutic potential warrants further mechanistic studies. Gaining a deeper knowledge of understudied tCDKs such as CDK8/CDK19, CDK 10, and CDK 11 could reveal their eligibility as therapeutic targets in multiple cancer types. Moreover, studies have started exploring the impact of tCDK inhibition on immunogenic signaling in cancer cells, probing them for sensitizing the innate immune system to enhance the efficacy of immunotherapy [110]. Furthermore, involvement of tCDK inhibitors in modulating anticancer immune cell populations in a tumor microenvironment warrants further detailed investigation [111].

## Data Availability

Not available.
